# Assessment of advanced paediatric dentistry education programmes in Egypt: a survey of programme directors

**DOI:** 10.1186/s12909-024-05176-w

**Published:** 2024-02-27

**Authors:** MK Yassa, NM Khattab

**Affiliations:** 1 Paediatric and Community Dentistry Department, Faculty of Dentistry, Minia University, Minia, Egypt; 2 Paediatric Dentistry and Dental Public Health Department, Faculty of Dentistry, Ain-Shams University, Cairo, Egypt

**Keywords:** Paediatric dentistry, Advanced education, Competency-based education

## Abstract

**Background:**

Advanced paediatric dentistry education programmes (APDEPs) should follow specific standards to produce competent specialists. The current study assessed APDEPs in Egypt via an online questionnaire to programme directors.

**Subjects and methods:**

An online questionnaire was distributed to the directors of fully operational degree-granting APDEPs in Egypt in June 2023. The survey instrument was based on the Accreditation Standards for Advanced Dental Education Programmes in Paediatric Dentistry developed by the Commission on Dental Accreditation (CODA).

**Results:**

Directors of the sixteen fully operational APDEPs answered the questionnaire giving a 100% response rate. APDEPs, in Egypt, varied regarding the adequacy of teaching staff, facilities and resources, didactic instruction, clinical requirements, and research activities.

**Conclusion:**

The current survey provides information about the strengths and weaknesses of fully operational degree-granting APDEPs in Egypt. This information can help maintain and improve the quality of these programmes.

## Introduction

Different from other specialties, paediatric dentistry deals with a specific group of patients rather than diseases or therapeutic areas. Treatment of children necessitates considering the physiological and psychological differences compared to adults [1].

Although the majority of dental services for children are performed by general dental practitioners, there are subgroups of children with additional needs such as very young age, handicapping conditions, anxiety, and extensive dental treatment needs that require paediatric dentistry specialists with higher competencies to deliver advanced and comprehensive oral health care [2].

To become a paediatric dental specialist, a dentist must complete an APDEP that provides didactic and clinical competency beyond the undergraduate level. APDEPs should follow specific standards to maintain and improve their quality. These standards are concerned with the educational didactic and clinical aspects, instructional activities, patient care, and the institution's facilities [3].

To the authors’ knowledge, no studies have assessed APDEPs, as a whole, in Egypt or any other country. Therefore, this study aimed to assess fully operational degree-granting APDEPs in Egypt via an online questionnaire to programme directors.

## Subjects and methods

The present study is a cross-sectional study in which an online questionnaire was distributed to the directors of fully operational degree-granting APDEPs in Egypt to provide survey-based analyses regarding programmes adherence to the standards of advanced dental education programmes in paediatric dentistry. This national survey followed the regulations of the Ethical Committee of the Faculty of Dentistry, Minia University, and ethical approval was obtained prior to conducting the study (reference no. 619/2022).

The survey instrument was based on the Accreditation Standards for Advanced Dental Education Programmes in Paediatric Dentistry developed by CODA [4].

The survey instrument comprised a brief introduction and 26-item questionnaire consisting of single and multiple-choice items, and rating scales to cover the following 6 areas recognized by CODA for an efficient programme;

1. Institutional commitment and programme effectiveness.
2. The director and teaching staff.
3. Facilities and resources.
4. Curriculum and programme duration.
5. Eligibility and selection, evaluation, due process, and rights and responsibilities.
6. Research.

Introduction, included in the survey instrument, intended to inform participants in an easy detailed manner about the objective of the study and to affirm that responses would be kept anonymous and results would be reported only as group data to ensure the respondents’ privacy and confidentiality.

Online survey software, was used. A preliminary survey instrument was sent to three paediatric dentistry professors to assess reliability, validity, and completion time and to identify components that needed clarification. Based on their suggestions about content and format, the survey instrument was modified, and the final version was developed. The survey was administered and APDEP directors were invited to complete the survey in June 2023. Reminders were sent on several occasions to those who did not complete the survey to increase participation. The survey was open for 8 weeks. Survey responses were collected into an Excel spreadsheet. Basic statistics and data analyses were performed using the Statistical Package for the Social Sciences software (version 20, SPSS, Chicago, Ill) for Windows 10.

## Results

Currently, fully operational and degree-granting APDEPs, in Egypt, are offered by nine faculties of dentistry. Seven of these faculties belong to governmental universities (Ain-shams, Alexandria, Cairo, Mansoura, Minia, Suez-canal, and Tanta universities) and two to private ones (the British University in Egypt and the Future University in Egypt). Although programmes offering the master's degree are provided by both governmental and private universities (*n*=9), doctor programmes are only provided by governmental universities (*n*=7). Programme directors were contacted and invited to participate in this survey to assess APDEPs. All directors responded giving an overall response rate of 100% (*n*= 16).

Survey data revealed the following:

1. Institutional commitment and programme effectiveness.

All programmes were sponsored by institutions accredited by the Supreme Council of Universities in Egypt/ Ministry of Higher Education and The National Authority for Quality Assurance and Accreditation of Education in Egypt.

2 Programme director and teaching staff.

All programme directors were certified in paediatric dentistry and educationally qualified, however, 50% of them were appointed to institutions with sufficient time and none of them had an annual evaluation. Regarding the teaching staff, only 50 % of programmes had an adequate number of teaching staff, and 81.25% of programmes provided efficient direct clinical supervision by the teaching staff.

Programmes exhibited differences regarding the teaching staff's responsibilities. The teaching staff was involved in curriculum development in four programmes (25%), use of technology in curriculum in ten programmes (62.5%), scholarly productivity in six programmes (37.5%), and presentations at paediatric dentistry meetings in twelve programmes (75%).

3 Facilities and resources provided by programmes.

**Fig 1:**
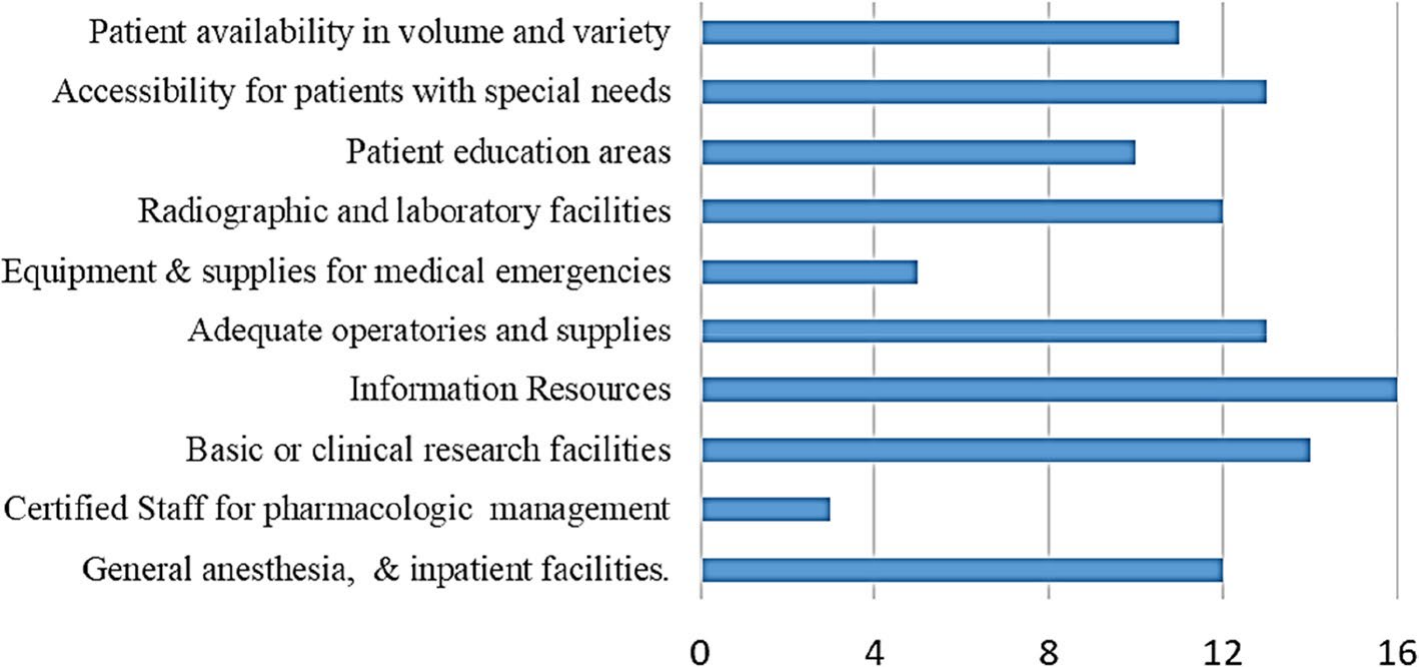
Facilities and resources provided by programmes

All programmes complied with regulations of national agencies regarding ionizing radiation hygiene and protection, hazardous materials, and blood-borne and infectious disease. Thirteen programmes (81.25%) provided easy access to the policies on bloodborne and infectious diseases while fourteen programmes (87.5%) encouraged students and staff to be immunized against and/or tested for infectious diseases. Variations in facilities and resources provided by programmes are summarized in Fig. 1.

4 Programme duration and curriculum.

Minimum programme duration ranged between two and three years in all programmes except one with four years. All programmes (100%) provided didactic and clinical skills exceeding undergraduate level and performed literature review seminars. However, 87.5% of programmes integrated evidence-based practice in didactic courses, while 75% of programmes encouraged searching databases and appraisal of the evidence, and required assignments for literature appraisal for best evidence.

**Fig 2:**
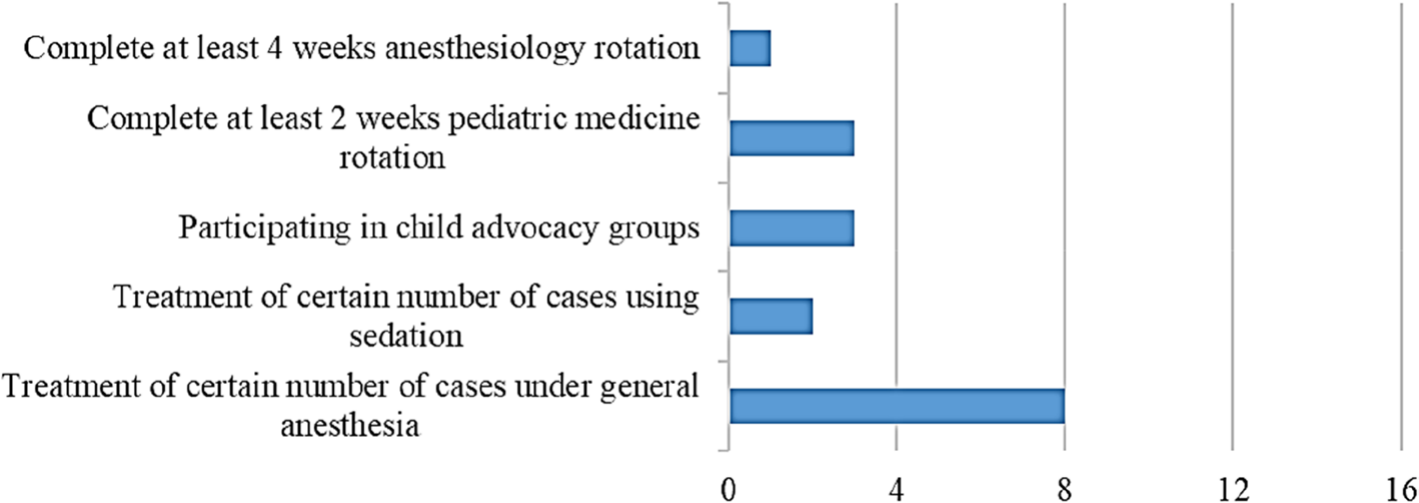
Programmes variations regarding their requirements

**Fig 3:**
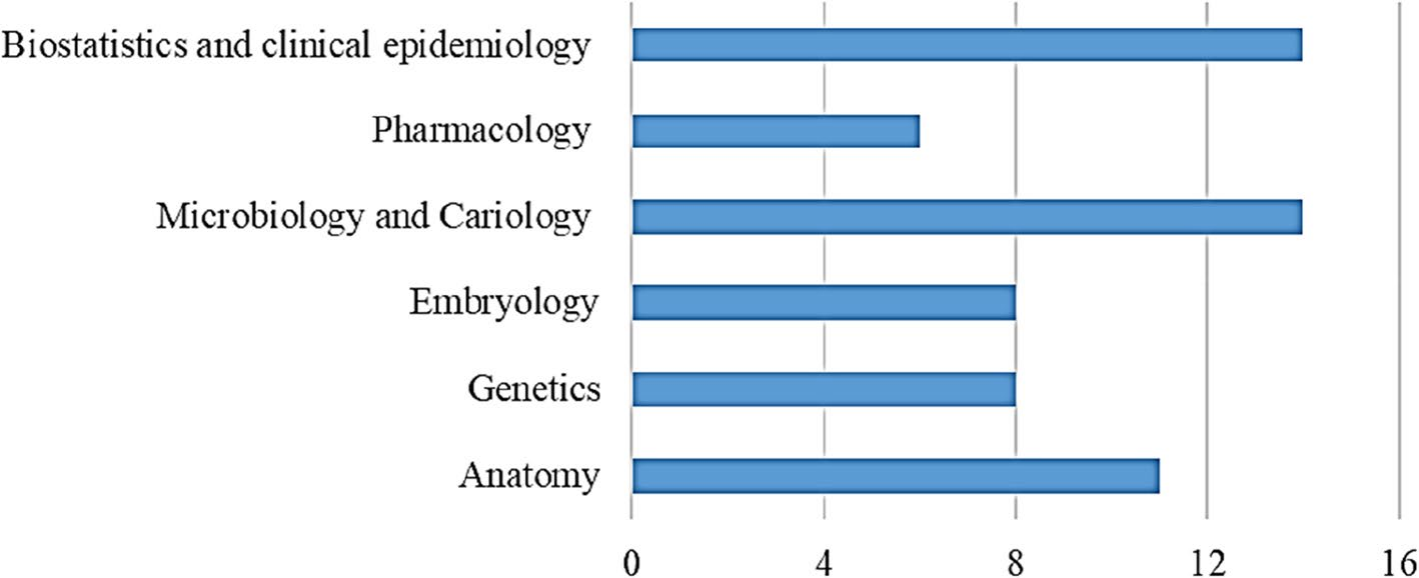
Programmes variations regarding knowledge in biomedical sciences

**Fig 4:**
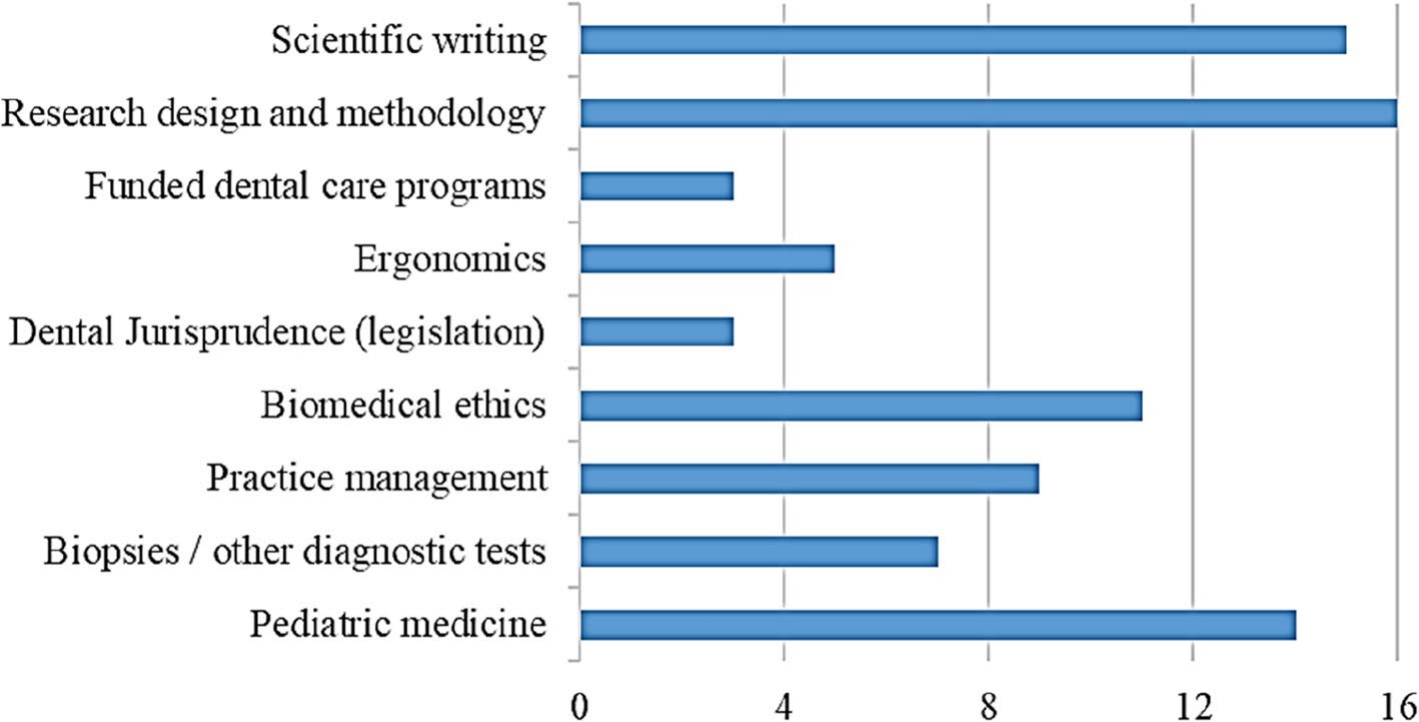
Programmes variations regarding didactic instruction at the understanding level

**Fig 5:**
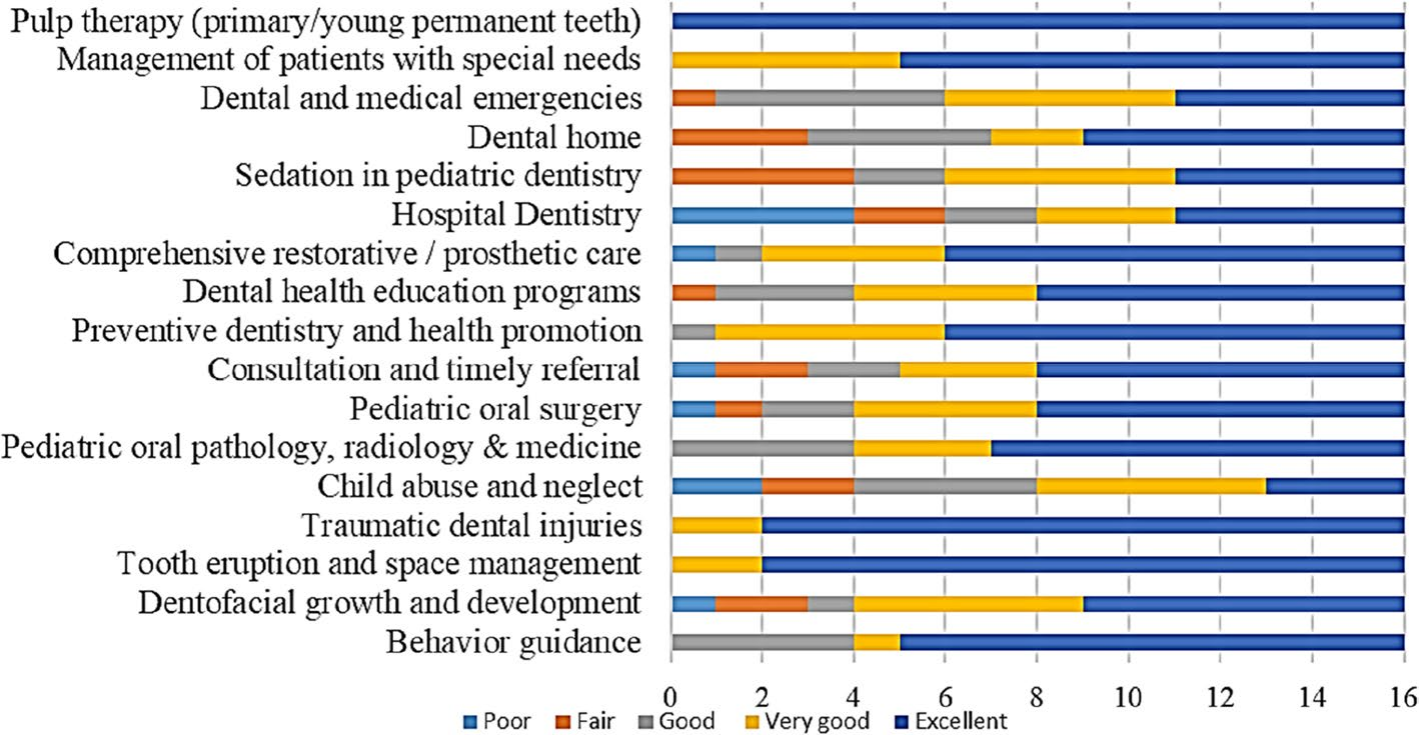
Programmes variations regarding the level of “in-depth didactic instructions” on a scale from 1:5 where 1 denotes poor and 5 denotes excellent

**Fig 6:**
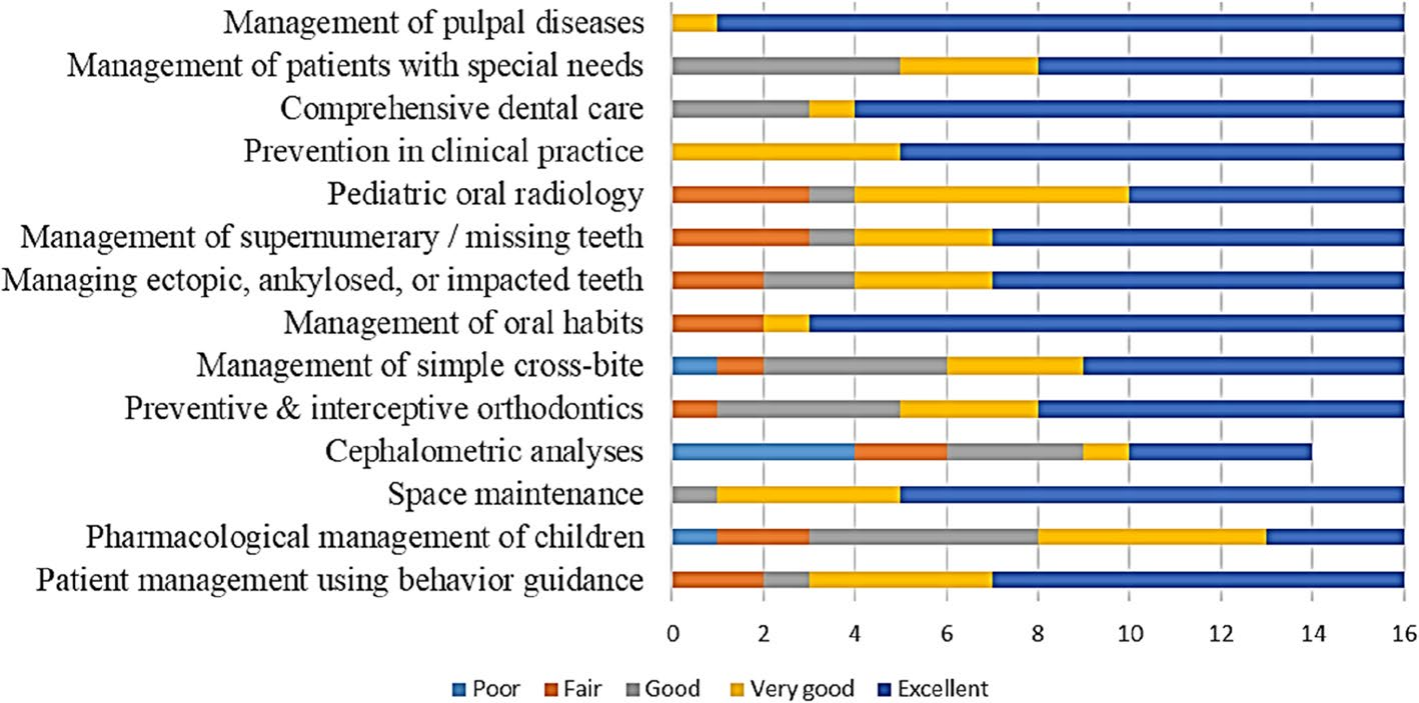
Programmes variations regarding the level of clinical experience to achieve competency on a scale from 1:5 where 1 denotes poor and 5 denotes excellent

Programmes exhibited variations regarding their requirements (Fig. 2), knowledge in biomedical sciences provided through formal courses or educational activities (Fig. 3), didactic instruction at the understanding level (Fig. 4), in-depth didactic instruction (Fig. 5), and clinical experience to achieve competency (Fig. 6).

5 Eligibility and selection, evaluation, due process, and rights & and responsibilities.

All programmes admitted students through specific written criteria, using non-discriminatory policies and eligible applicants had to be graduates from the accredited undergraduate programme. Fourteen programmes (87.5%) provided students with written information on the programme which affirmed their obligations and responsibilities to the university, the faculty, and the programme. Students’ evaluation was at least semi-annually in eleven programmes (68.75%) and an external examiner had to be consulted as a part of the final examination in fifteen programmes (93.75%).

6 Research

Although candidates in all programmes had to complete a research project as a programme requirement, candidates in nine programmes (56.25%) had to report project results in a scientific forum and candidates in eleven programmes (68.75%) had to publish a paper in an indexed journal. Data collection and analysis were requirements in ten programmes (62.5 %).

## Discussion

Untreated carious lesions in primary teeth affect more than 40 million Arabian children, while in permanent teeth, the unmet need exceeds 137 million. Global trends show that low and middle-income countries, including Egypt, have the highest burden of dental caries [5]. In 2014, the Egyptian Ministry of Health, in collaboration with the World Health Organization (WHO) country office, released the results of a nationwide oral health survey declaring that nearly 70% of examined children had some untreated carious lesions [6].

This high unmet need for dental services coupled with the accelerating growth of the Egyptian population, which is expected to reach 120 million by 2030, and the high prevalence of people with special health care needs that exceeds 10% of the population place more emphasis on providing sufficient paediatric dentistry knowledge and training to meet the high national oral health care needs [7–9].

The current study assessed the quality of APDEPs in Egypt via an online survey submitted to programme directors. APDEPs need to respond to innovative curricula, enhanced education, and training methods to produce specialists who are competent in all areas of the specialty [10, 11].

The survey instrument was based on the accreditation standards for APDEPs developed by CODA which is recognized by the United States Department of Education as the specialized accrediting agency. The CODA recognized six areas for an efficient programme and defined items under each area. These standards are comprehensive valid items for quality assurance [4].

The survey instrument comprised close-ended questions in the form of Yes/No questions, multiple choice, and scaled questions since the range of answers was known and limited to a fixed set of responses. A preliminary survey instrument was sent to three paediatric dentistry professors to determine content validity via their expert judgment, and for pretesting. Based on their suggestions, the survey instrument was developed and an online survey was conducted to avoid interviewer bias and distortion [12].

The current survey was limited to the directors of degree-granting APDEPs, in Egypt, that had a planned sequence of advanced courses leading to a master’s or doctoral degree granted by an accredited educational institution. In addition, included programmes were fully operational that graduated at least one class of students/residents and had students/residents enrolled in each year of the programme. Currently, there are sixteen fully operational degree-granting APDEPs in Egypt. These programmes conformed to the CODA standards regarding the programmes minimal duration that mandate at least two years. Only two out of the sixteen programmes belonged to private dental schools since private schools are for-profit businesses that prefer to admit a higher number of undergraduate students than the limited postgraduate students [11].

All the invited directors (*n*=16) participated in the survey (100% response rate). Ericson et al. (2023) [13] reported that a high response rate can be achieved when a relationship between the survey administrator and recipients exists, a condition that was provided by the authors. Another driver for the high response rate is the common interest in the research. A high response rate enables production of generalizable data that are crucial in dental education survey research [14].

Programme directors and teaching staff demonstrated good standing in terms of education and educating their profession. However, the teaching staff was inadequate in number and they were appointed to the institution with insufficient time that decreased their involvement in direct clinical supervision, curriculum development, and scholarly productivity. The United Kingdom Committee of Postgraduate Dental Deans and Directors (COPDEND UK, 2013) [15] stressed that teaching staff commitments include both teaching and educational leadership responsibilities.

Facilities and resources enable dental students to acquire the essential competency [16]. Studied programmes exhibited evident shortages in the licensed deep sedation/general anesthesia staff and the equipment and supplies for medical emergencies. Efforts should be exerted to overcome this shortage since oral health has to be placed within the context of systemic health [17, 18].

APDEPs, in Egypt, varied in the degree of knowledge in biomedical sciences, didactic instruction, integrating evidence-based practice, clinical requirements, and clinical experience to achieve competency. Competency can be described simply as the personal ability of a professional [19]. The current results revealed that more efforts should be exerted to expose candidates to advocacy strategies and legislative processes. Moreover, there is variability among programmes regarding research. Interference with clinic times, lack of faculty interest in research, and lack of financial resources are the major obstacles to postgraduate research [20].

Many surveys have evaluated the opinions of APDEP directors regarding selected areas of training such as sedation training [21], atraumatic restorative treatment [22], silver diamine fluoride, and Hall technique [23]. Moreover, other surveys have evaluated areas concerning the education process such as the need for alternative didactic learning options [24], and evaluation of the assessment techniques [25]. However, up to the authors’ knowledge, this is the first survey of its kind that evaluates the entire APDEP shedding light on the limitations of each programme.

This survey instrument can be used as a multidimensional assessment tool to assist stakeholders and programme directors in evaluating and improving existing programmes and structuring new ones since high-quality education is essentially linked to high-quality health care [26].

However, a limitation of the study is the self-reporting nature of the data depending totally on APDEP directors’ opinions that might have induced unintentional bias. Al Achkar et al. (2018) [27] reported that programme directors may not be fully aware of the details within their programs. Some programme directors may overestimate how much postgraduate students receive, whereas others likely underestimate this. Rutkauskas et al. (2015) [28] and Sheets et al. (2016) [29] have reported a disparity in perceptions between residents and programme directors. To bridge this gap, it is recommended to execute a similar nationwide survey of paediatric dentistry specialists to assess their perceptions regarding their APDEPs.

## Conclusion

The current survey provides information about the strengths and weaknesses of fully operational degree-granting APDEPs in Egypt. This information can help maintain and improve the quality of these programmes.

## Data Availability

All data generated during this study are included in this article.

## Abbreviations

APDEPs: Advanced paediatric dentistry education programmes
CODA: Commission on Dental Accreditation
CAPMAS: Central Agency of Public Mobilization and Statistics
COPDEND UK: The United Kingdom Committee of Postgraduate Dental Deans and Directors
